# Double yolk eggs detection using fuzzy logic

**DOI:** 10.1371/journal.pone.0241888

**Published:** 2020-11-05

**Authors:** Thanasan Intarakumthornchai, Ramil Kesvarakul

**Affiliations:** 1 Department of Industrial Engineering, Faculty of Engineering, King Mongkut’s University of Technology North Bangkok, Bangkok, Thailand; 2 Department of Production Engineering, Faculty of Engineering, King Mongkut’s University of Technology North Bangkok, Bangkok, Thailand; Hefei University of Technology, CHINA

## Abstract

Chicken egg products increased by 60% worldwide resulting in the farmers or traders egg industry. The double yolk (DY) eggs are priced higher than single yolk (SY) eggs around 35% at the same size. Although, separating DY from SY will increase more revenue but it has to be replaced at the higher cost from skilled labor for sorting. Normally, the separation of double yolk eggs required the expertise person by weigh and shape of egg but it is still high error. The purpose of this research is to detect double-yolked (DY) chicken eggs with weight and ratio of the egg’s size using fuzzy logic and developing a low cost prototype to reduce the cost of separation. The K-means clustering is used for separating DY and SY, firstly. However, the error from this technique is still high as 15.05% because of its hard clustering. Therefore, the intersection zone scattering from using the weight and ratio of the egg’s size to input of DY and SY is taken into consider with fuzzy logic algorithm, to improve the error. The results of errors from fuzzy logic are depended with input membership functions (MF). This research selects triangular MF of weight as low = 65 g, medium = 75 g and high = 85 g, while ratio of the egg is triangular MF as low = 1.30, medium = 1.40 and high = 1.50. This algorithm is not provide the minimum total error but it gives the low error to detect a double yolk while the real egg is SY as 1.43% of total eggs. This algorithm is applied to develop a double yolk egg detection prototype with Mbed platform by a load cell and OpenMV CAM, to measure the weight and ratio of the egg respectively.

## Introduction

Among commercial species of poultry, double yolk (DY) eggs are seen frequently. Typically, an oocyte is discharged by a hen every 18–26 hours, from the left ovary into the oviduct. While adding layers of the egg along the path to the vent where lays the egg, the oocyte moves across the oviduct organ. When two oocytes ovulate in the same 3-hour period and converge into the same egg, a DY egg is created [1,2]. In broiler breeder pullet eggs during the first three months of egg-laying, this occurs 4~12.5% of the time [3,4]. In laying hen eggs, it is estimated to occur 1.1~3.5% of the time [5].

Because of the comparatively low fertility rate, DY eggs are deemed a loss to commercial hatching compared to single yolk (SY) eggs. This is due to the smaller yolk and the perceptibly reduced hatching rate as a result of less space for movement [6–8]. Contaminating other hatching eggs by bacterial or fungal infection as well as lost space and power in the incubator are risks of these eggs. Thus, a commercial hatchery will separate most DY eggs prior to hatching [9,10]. On the other hand, some countries regard DY eggs as a symbol of good luck and use the DY eggs as a greeting in various festivals. In order to increase the value of DY eggs, it could be advantageous if the farmer could separate the DY eggs for sale in another market segmentation.

A conventional technique called “egg candling” is done by conducting light through them to identify DY eggs. An inspector is able to see the yolk using this method in order to determine if it is a SY or DY egg. A computer vision system to detect DY eggs was created by Wang et al. [11]. As a way to detect DY eggs with more than 95% accuracy, geometrical features from digital images gained during egg candling were used. Including the egg’s area, yolk area to perimeter ratio, yolk area to egg area ratio, and yolk perimeter to the egg’s perimeter ratio, the geometrical features of the egg were used to assess the criteria. The egg’s area is used to regulate all factors. Actually, some duck eggs are nearly the same size as SY eggs, which are produced all the time. This is why the system makes decision errors. Methods to separate DY from SY duck eggs using computer vision with Fisher’s linear discriminant (FLD) and convolutional neural network (CNN) models were created by Ma et al. [12]. The geometrical characteristics from the digital images from the egg candling process were used to extract the yolk shape. In the image preparation process, thresholding was used to convert the RGB image into a binary image. Different lighting intensity was used in the thresholding value. Therefore, the algorithm may make a wrong decision if using the improper thresholding value. However, these technique are mostly used for duck eggs and required more equipment and setting. Therefore, the separating of DY of chicken eggs with easy use and low cost are examined.

This study investigated the potential of using the weight and ratio of the egg’s size to input with fuzzy logic to classify chicken eggs into DY categories and applied this algorithm to DY detection machine prototype with Mbed platform by measuring the weight with load cell, size of the egg with OpenMV CAM and processing with Arduino.

## Double yolk egg indicator

Since eggs are of varying sizes, it is difficult to correctly classify DY eggs by analyzing just one size or weight. From interview of the experts to sort DY eggs, they decision it by geometry and weight of the eggs, it was found that DY eggs would have a ratio of length to the width rather than SY eggs and had a lot of weight. However, they could still not exactly determine which of the above reasons would be considered as an egg with more than one egg yolk. This was because in some cases the DY eggs had a proportion of length per width and weight similar to SY eggs.

**Fig 1:**
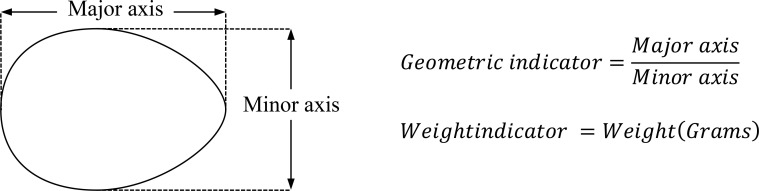
Geometric indicator. The data are divided it into two indicators; namely, the geometric indicator and weight indicator. The geometric indicator was the ratio of length to the width of the egg. The second indicator was the weight indicator, which was the weight of the egg in grams.

The researchers collected the data by dividing it into two indicators; namely, the geometric indicator and weight indicator. The geometric indicator was the ratio of length to the width of the egg; i.e., the ratio between the major axis length and minor axis length (refer to Fig 1). The second indicator was the weight indicator, which was the weight of the egg in grams.

**Fig 2:**
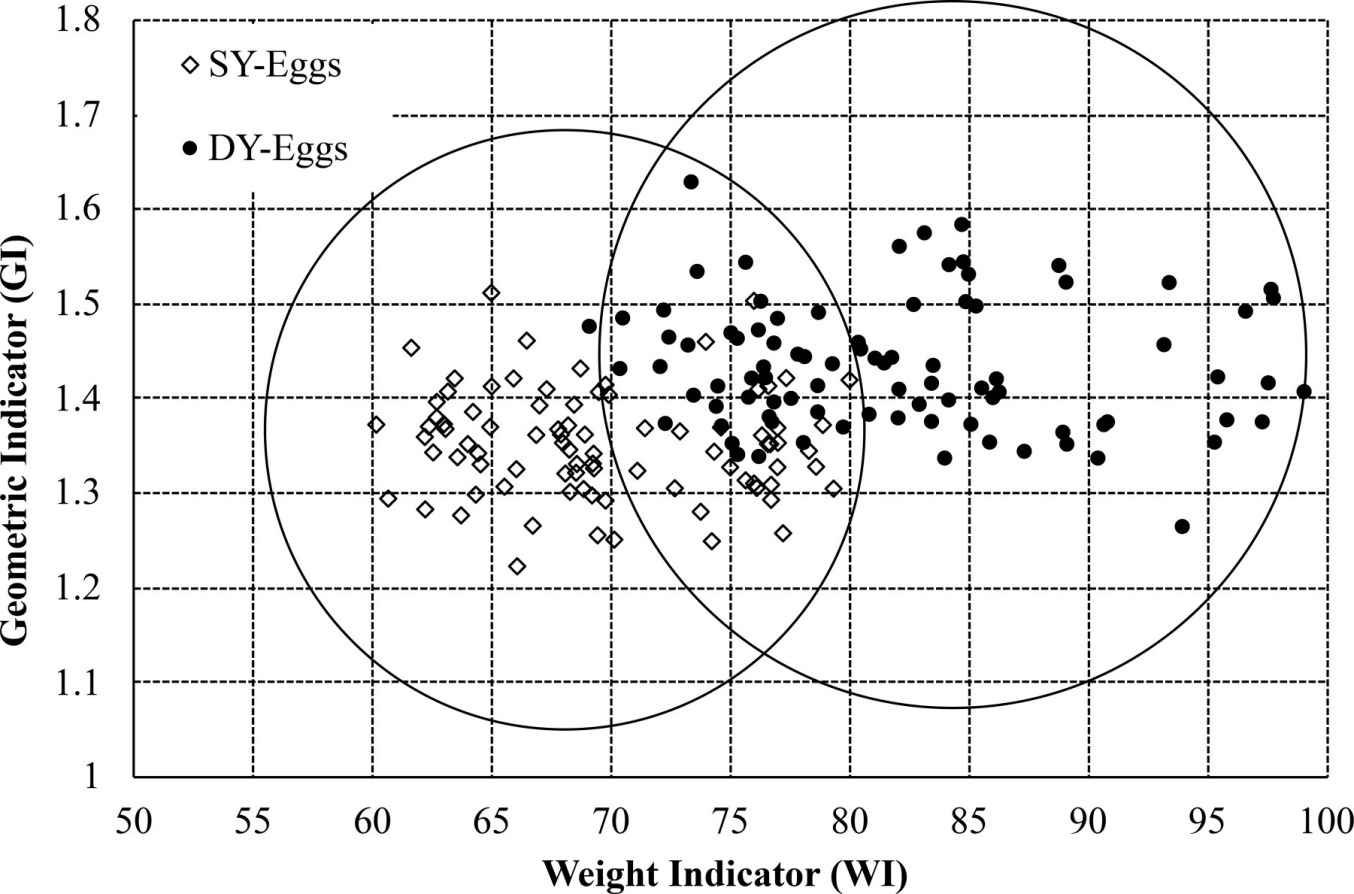
Relationship between the geometry indicator and weight indicator. The group was classified into two clusters. The left cluster was clearly classified that there was a SY egg cluster. The right cluster was clearly classified as a DY egg cluster. The intersection area was a sample with a fuzzy member that could not be clearly identified as DY eggs or SY eggs.

A total of 99 DY and 180 SY chicken eggs from a Saha Farms Company limited in Thailand using in experiment were identify by broken them and obvious. Fig 2 shows the relationship between the geometry indicators and weight indicators. Thus, it was found that the DY eggs weighed over 70 grams, and the scatter plot was separated into two clusters. The first cluster of DY eggs was a large egg that could be easily classified. On the other hand, the DY eggs of the second cluster were small eggs with a data distribution similar to large SY eggs. Therefore, the group was classified into three clusters. The left cluster was clearly classified that there was a SY egg cluster. The right cluster was clearly classified as a DY egg cluster. The center cluster was a sample with a fuzzy member that could not be clearly identified as DY eggs or SY eggs.

## K-means clustering

Clustering techniques are valuable tools for due with large database. K-means clustering is a method commonly used to automatically partition a data set into k groups [13]. In this research, 279 chicken eggs are measure the weight by scales and the major and minor axis length by Vernier caliper to calculate the ratio (refer to Fig 1). These data points are in 2-dimensional space weight and ratio. This problem is to determine the center of 2 groups (k = 2) by following procedure:

1. Random the initial centroids of 2 groups
2. Calculate the distance between points to centroids
3. Separate points into 2 groups by minimum distance from centroid
4. Calculate the new centroids by average (means) of points in its group
5. Iteration step 2–4 until the new centroids do not change

The result of centroids in each iteration was shown in Table 1. The 7^th^ iteration was performed the optimum centroid of C1 (SY) as 69.61 g of weight and 1.35 of ratio and the centroid of C2 (DY) as 84.92 g of weight and 1.42 of ratio.

**Table 1:** K-means iterations.

	Centroid C1		Centroid C2	
	Weight	Ratio	Weight	Ratio
Initial	57.6400000	1.25990000	97.78000	1.506500
Iteration 1	69.7996744	1.34968245	85.50016	1.424880
Iteration 2	69.7627103	1.35011116	85.38031	1.422312
Iteration 3	69.7257746	1.35029279	85.14642	1.420498
Iteration 4	69.6956132	1.34986939	85.12642	1.420922
Iteration 5	69.6520379	1.34976431	85.03471	1.420203
Iteration 6	69.6153810	1.34942157	84.92333	1.420225
Iteration 7	69.6153810	1.34942157	84.92333	1.420225

**Fig 3:**
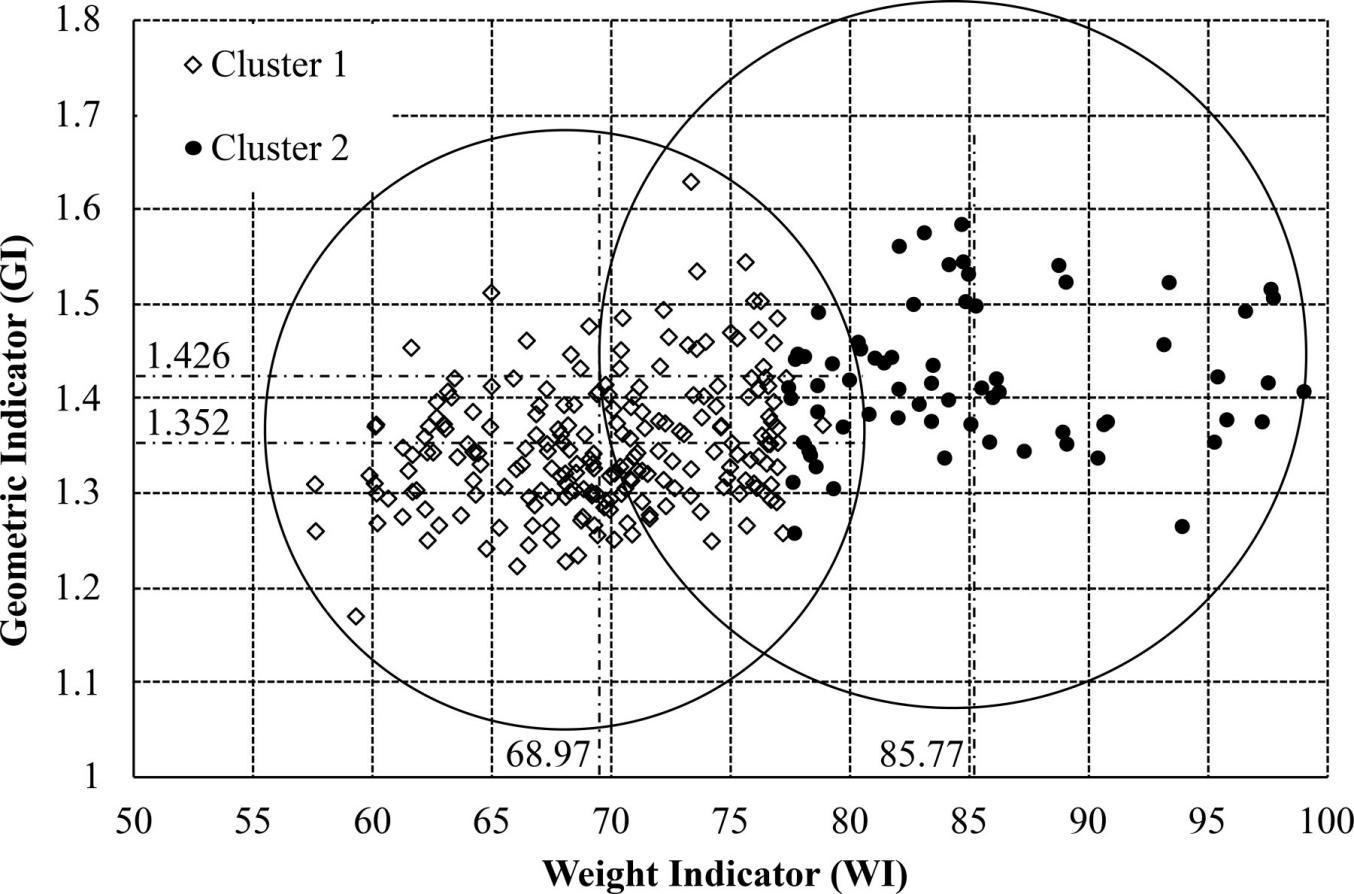
The points of SY and DY group in final iteration. The number of SY by K-means method were 210 eggs while the number of DY were 69 eggs.

The points of SY and DY group in final iteration were shown in Fig 3. The number of SY by K-means method were 210 eggs while the number of DY were 69 eggs. This technique showed the single yolk error (a cracked egg was single while the algorithm showed a double yolk egg) as 11.11% and the double yolk error (a cracked egg was double while the algorithm showed a single yolk egg) as 3.94%, so the total error was 15.05%. The overall errors were higher enough due to this technique determines the center of group by distributing the data around the center uniformly and there is not intersection area because of a hard clustering. While the data from Fig 2 show DY and SY are scatter not uniform and there is an intersection area between DY and SY. Therefore, K-means technique is not proper for clustering the DY from SY. The soft clustering such a fuzzy logic is taken into account in the next section.

## Fuzzy logic algorithm

The principles of approximate reasoning and computational intelligence form the basis for fuzzy logic research. In situations where it may be hard for a system to have an exact model (though an imprecise model may exist) or uncertainty and inaccuracy are met during the problem formulation, fuzzy models are used. Linguistic variables (e.g., low, medium, and high), and an uninterrupted range of truth values in the interval [0, 1] [14] are used as well.

The fuzzy control area has obviously been the most effective application area for fuzzy systems. Ordinarily, fuzzy controllers are distinct expert systems. Conveyed in terms of relevant fuzzy inference rules and a suitable inference engine to solve a given controlled problem, each uses a knowledge center. As a substitute to a precise model of the controlled process, a qualified human operator’s knowledge may be employed. An inexact linguistic account of the manner of control can typically be expressed by an operator with comparative ease, even when the knowledge is difficult to convey in specific terms. This linguistic account contains a set of control rules that employ fuzzy propositions. A characteristic form of these rules can be described using the rule:

IF the geometric indicator is high,
AND the weight indicator is medium
THEN the eggs are double yolk.

GI and WI varying is the act taken by the controller when a single yolk or double yolk egg is seen during the classification process. Described in the space of this course in terms of the geometric indicator and weight indicator, the indistinct terms *low*, *medium* and *high* can be usefully shown by fuzzy sets. The foundation for the concept of many fuzzy controllers is formed by this kind of linguistic rule.

**Fig 4:**
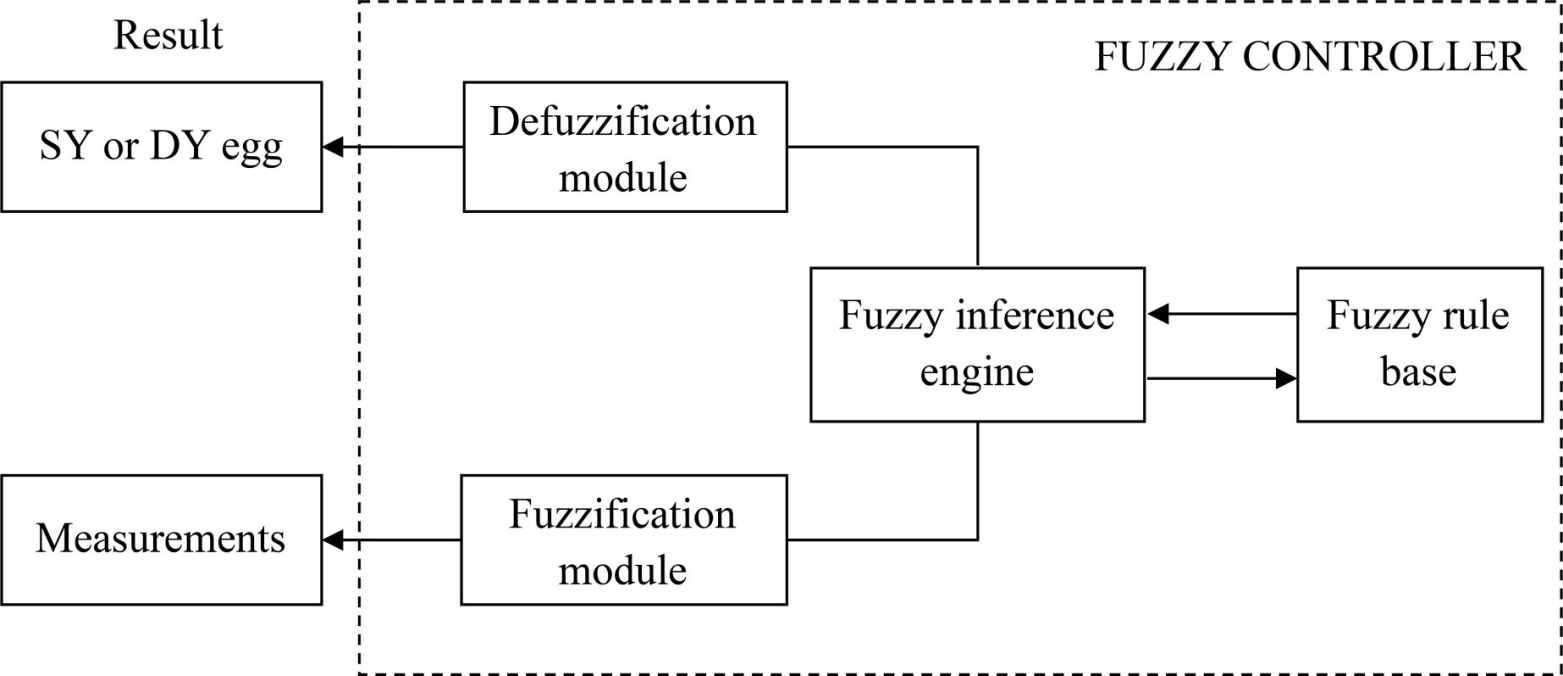
A fuzzy controller scheme of DY and SY classification. The links between four modules comprising a fuzzy controller, including a *fuzzy rule base*, a *fuzzy inference engine*, and *fuzzification/defuzzification*.

There are typically four modules comprising a fuzzy controller, including a *fuzzy rule base*, a *fuzzy inference engine*, and *fuzzification/defuzzification*. Fig 4 shows the links between these modules and the control process.

### Membership function

A curve that describes how each point in the input space is charted to a membership value (or degree of membership) between 0 and 1 is called a membership function. To resolve their impact on the fuzzy output sets of the deduction of the final output, the input membership values are operated as ratio and weighting factors in the fuzzy rules. The functions are defuzzified into a crisp output, which compels the control system, after they are implied, scaled, and joined. Therefore, the membership function is an important factor for giving the accuracy of the system responses.

**Fig 5:**
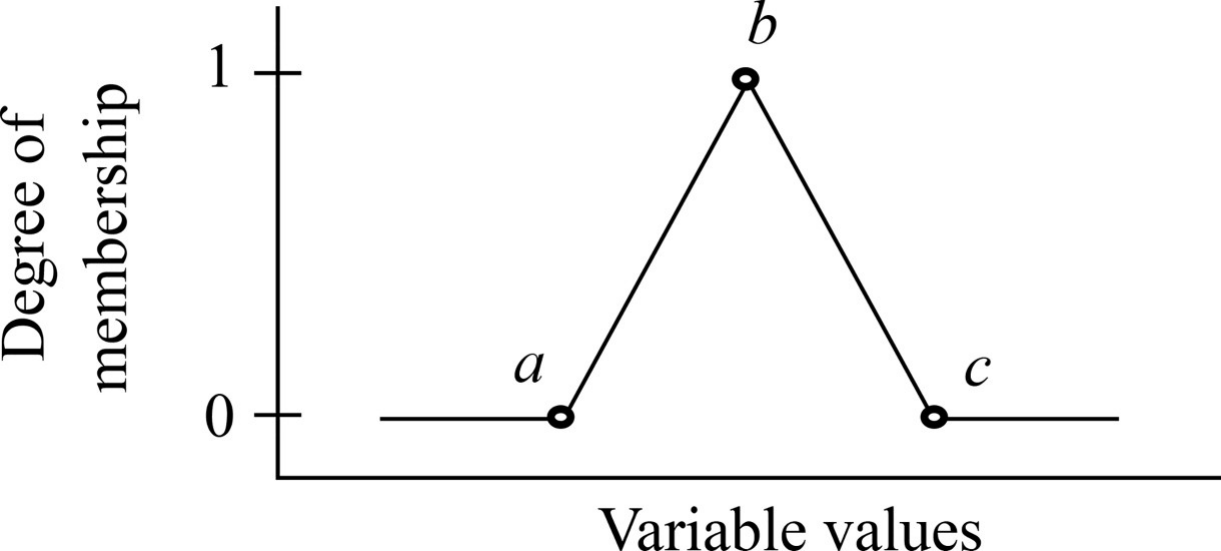
The full triangular membership function.

A triangular shape is applied for all input and output variables. A full triangular membership function (Trimf) is shown in Fig 5 and Eq (1).

$$Trimf(x:a,b,c)=\left\{ \begin{array}{l} \;0\;;x\leq a\\ (x-a)/(b-a)\;;a<x<b\\ \;1\;;x=b\\ (c-x)/(c-b)\;;b<x<c\\ \;0\;;x\geq c \end{array}\right.$$ (1)

**Fig 6:**
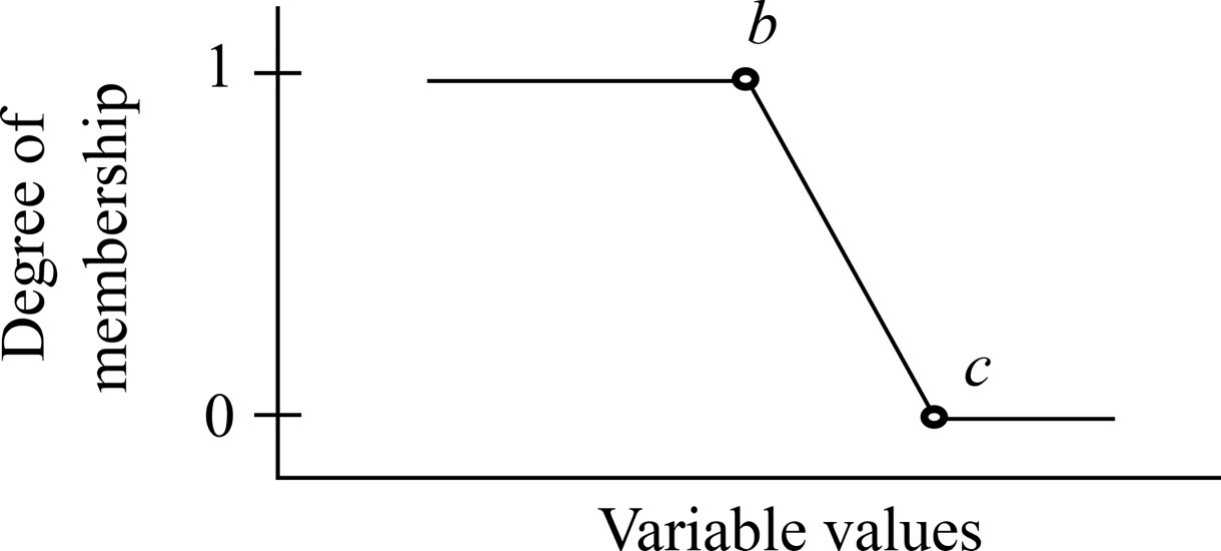
The half triangular membership function (Right-hand side).

**Fig 7:**
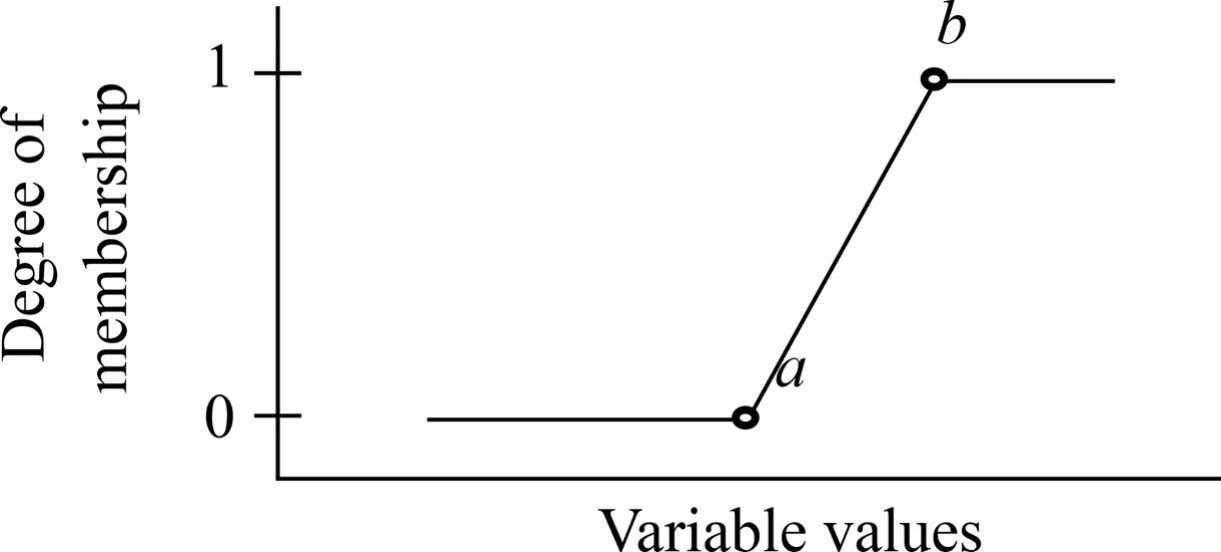
The half triangular membership function (Left-hand side).

The halves of the triangular shape have two sides: the right-hand side (refer to Fig 6 and Eq (2)) and the left-hand side (refer to Fig 7 and Eq (3)).

$$Trimf(x:a,b,c)=\left\{ \begin{array}{l} \;1\;;x\leq b\\ (c-x)/(c-b)\;;b<x<c\\ \;0\;;x\geq c \end{array}\right.$$ (2)

$$Trimf(x:a,b,c)=\left\{ \begin{array}{l} \;0\;;x\leq a\\ (x-a)/(b-a)\;;a<x<b\\ \;1\;;x\geq b \end{array}\right.$$ (3)

**Fig 8:**
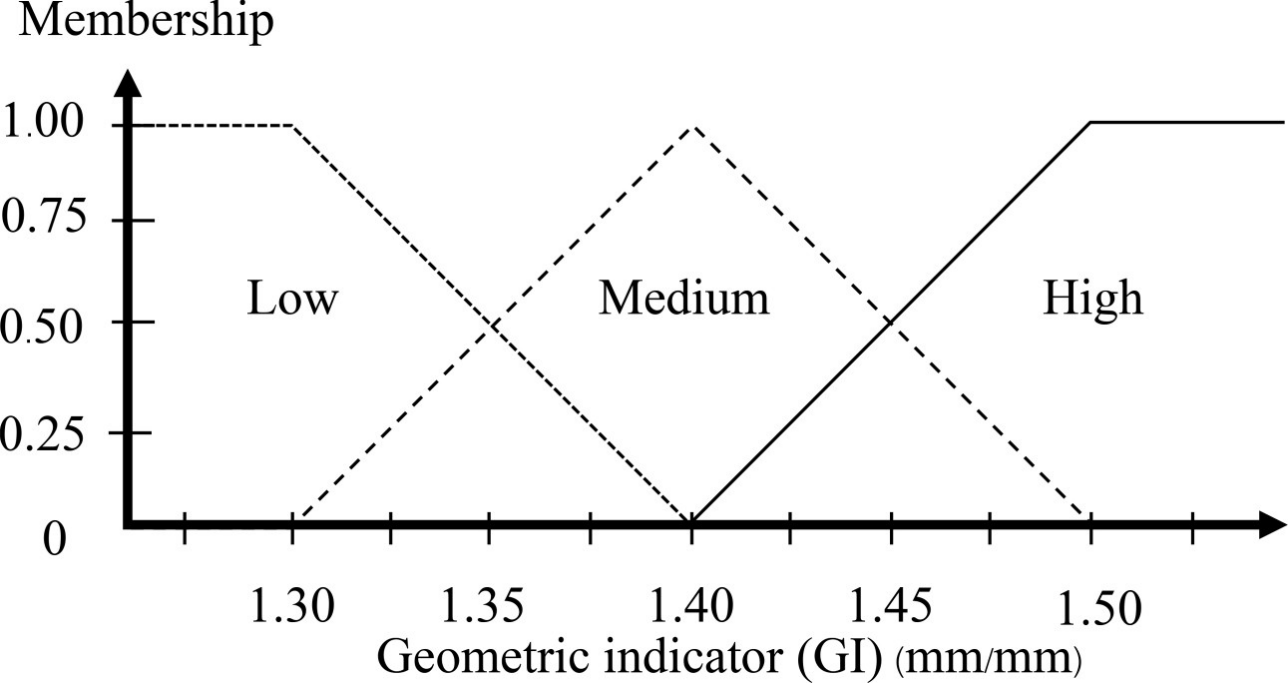
Input membership functions of the geometric indicator.

In Fig 3, the ratio of the egg is in the range around 1.30–1.50. A higher ratio than 1.50 is high that means it is assured to be a DY egg. The ratio of the egg as 1.30 is defined as the minimum value of the GI membership function. For an equally spread triangular shape, the middle values as 1.40 are assured to be medium. The high value and low value are defined with a half triangular membership function, and the medium value is defined as a full triangular membership function. Fig 8 displays the GI membership function.

**Fig 9:**
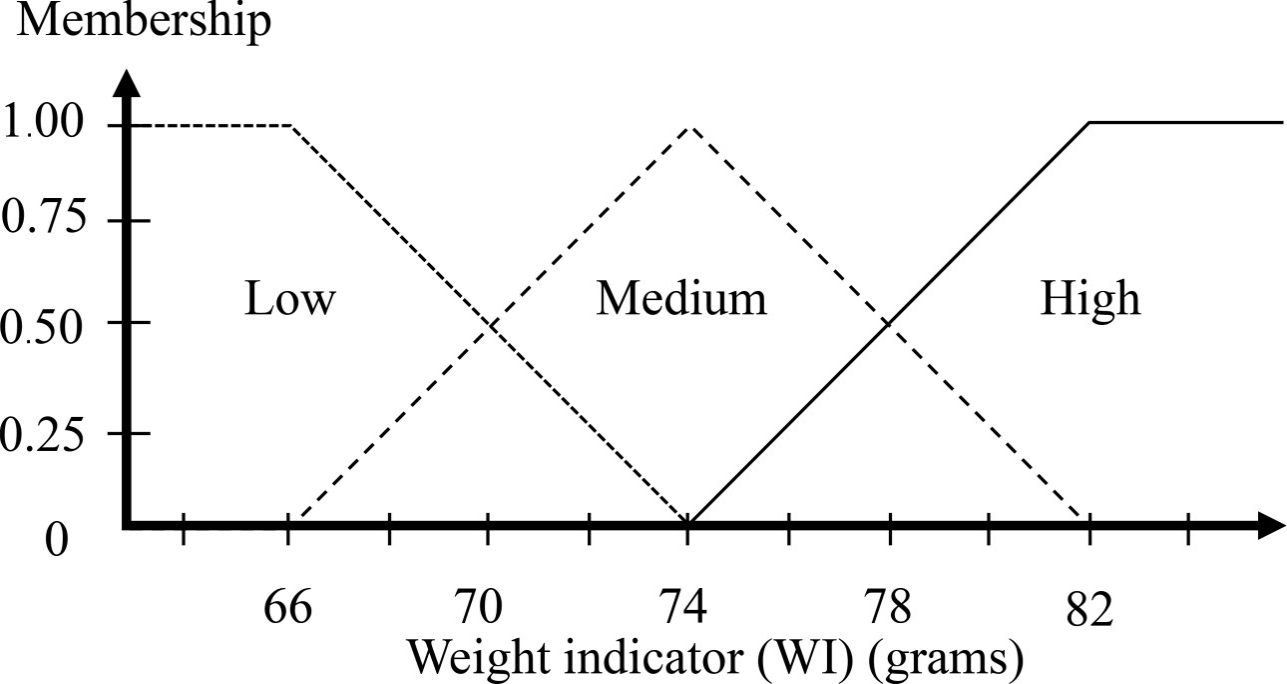
Input membership functions of the weight indicator.

The typical weight is in the range of 55 to 100 grams. More than 82 grams is likely to result in a large egg (DY egg), so it has a high weight if the weight indicator is more than 82 grams. A weight less than 66 grams is clearly a low weight, as this is the maximum value found in a SY egg. The middle value of the range as 74 grams is obviously medium. The high value and low value are defined with a half triangular membership function, and the medium value is defined with a full triangular membership function. Fig 9 is the WI membership function.

**Fig 10:**
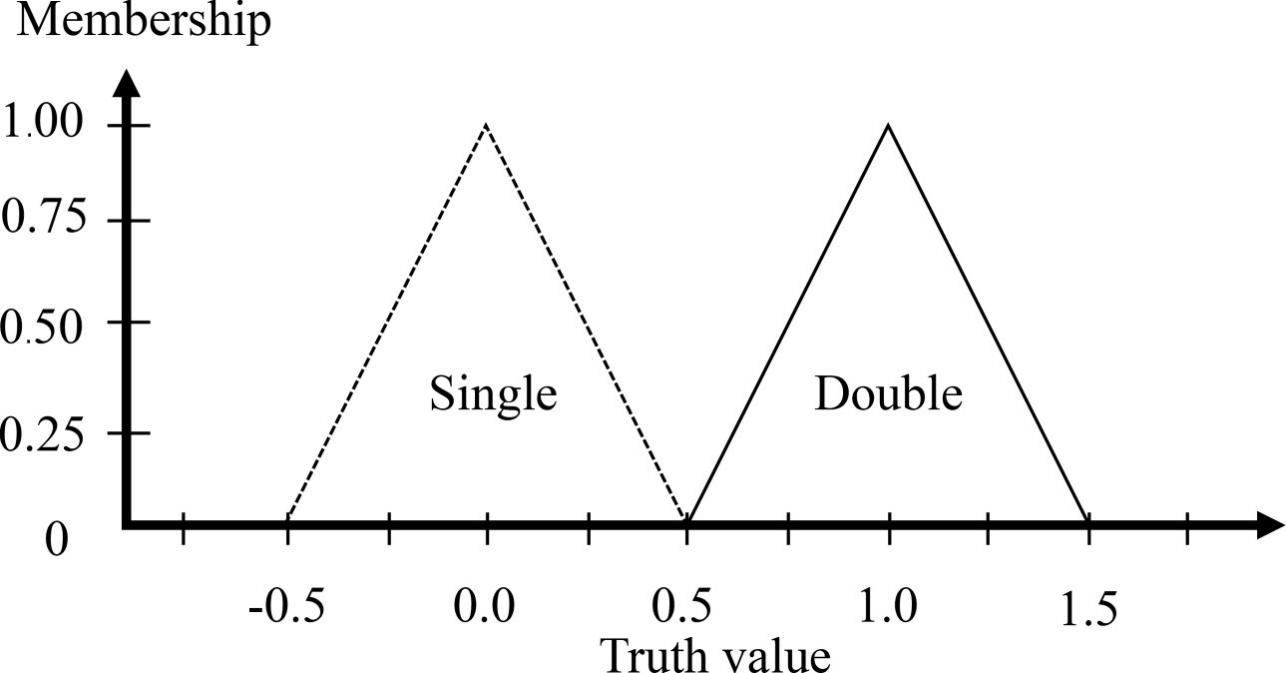
Output membership functions of the truth value.

The output of the double yolk egg detection is the truth value that means a probability to detect double yolk eggs. The truth value membership function is defined as full triangular, the center of a single value (fault) is 0, and the center of a double value (true) is +1 (refer to Fig 10).

### Rules

The operator’s capability and knowledge of the experiment from gauging the ratio and weight of the eggs are typically used to obtain the fuzzy rules to be used prior to including the fuzzy rules into the DY egg detection system. For the geometry indicator and weight indicator, the input variables are divided into three classes as low, medium and high. Used to assess if eggs are DY or SY, these inputs stimulate nine fuzzy logic rules. Subsequently, the output variables as the root sum square of the output set are calculated using a defuzzification process. The whole set of collected rules are show as follows and in Table 2. where (0) and (1) is the eggs have single yolks and the eggs have double yolks respectively.

Rule 1: If the geometric indicator is low and weight indicator is low, the eggs have single yolks. (0)
Rule 2: If the geometric indicator is low and weight indicator is medium, the eggs have single yolks. (0)
Rule 3: If the geometric indicator is low and weight indicator is high, the eggs have double yolks. (1)
Rule 4: If the geometric indicator is medium and weight indicator is low, the eggs have single yolks. (0)
Rule 5: If the geometric indicator is medium and weight indicator is medium, the eggs have single yolks. (0)
Rule 6: If the geometric indicator is medium and weight indicator is high, the eggs have double yolks. (1)
Rule 7: If the geometric indicator is high and weight indicator is low, the eggs have single yolks. (0)
Rule 8: If the geometric indicator is high and weight indicator is medium, the eggs have double yolks. (1)
Rule 9: If the geometric indicator is high and weight indicator is high, the eggs have double yolks. (1)

**Table 2:** Fuzzy logic rules for the DY eggs detection system.

		Weight Indicator		
		Low	Medium	High
	Low	R1 (0)	R2 (0)	R3 (1)
Geometry Indicator	Medium	R4 (0)	R5 (0)	R6 (1)
	High	R7 (0)	R8 (1)	R9 (1)

### Fuzzication

**Fig 11:**
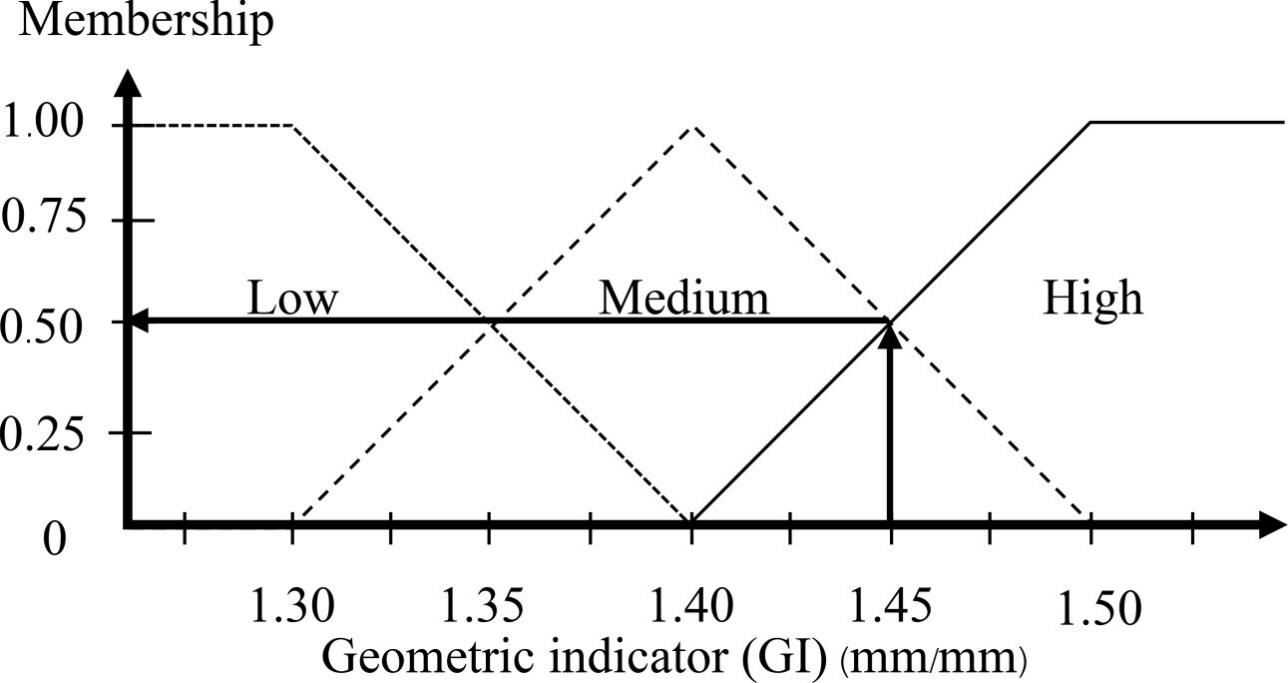
Input of the geometric indicator is 1.45 mm.

**Fig 12:**
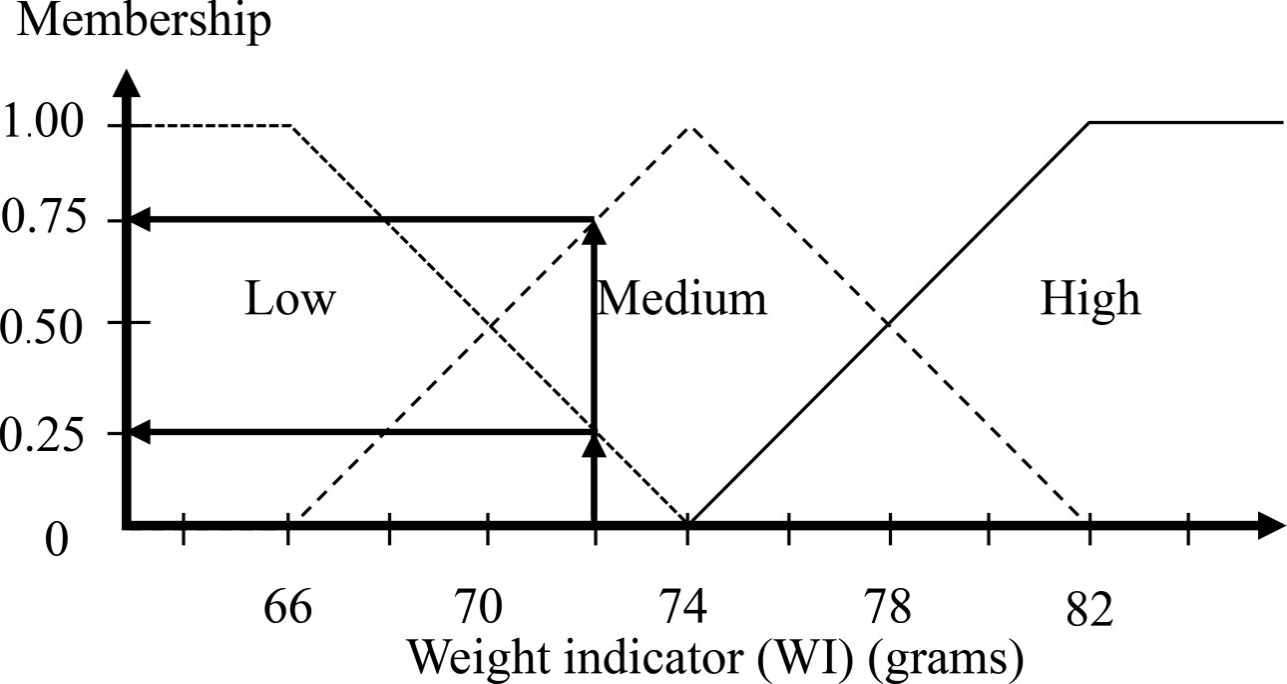
Input of the weight indicator is 72 grams.

The step after the inputs are fuzzified involves establishing the degree that each part of the antecedent is satisfied for each rule. The fuzzy operator is used to get one number that signifies the result of the precursors for that rule if the precursor of a given rule has multiple parts. The number can then be used in the output operation. Two or more membership values from the fuzzified input variables comprise the input to the fuzzy operator (refer to Figs 11 and 12). It is understood that the ratio of the egg is 1.45 mm and the weight of egg is 72 grams as the output is a single truth value and methods are reinforced: *min* (minimum). For each input variable, identify the degree of the fuzzy memberships using:

Geometric indicator (GI): Low = 0.00, Medium = 0.50, High = 0.50
Weight indicator (WI): Low = 0.25, Medium = 0.75, High = 0.00

Based on the input memberships, nine cases (*R*1–*R*9) of situations exist, and the fuzzy operation for all rules is done. The probabilistic situation and method develops the subsequent resulting details:

Rule 1: Min (GI, WI) = Min (0.00, 0.25) = 0.00
Rule 2: Min (GI, WI) = Min (0.00, 0.75) = 0.00
Rule 3: Min (GI, WI) = Min (0.00, 0.00) = 0.00
Rule 4: Min (GI, WI) = Min (0.50, 0.25) = 0.25
Rule 5: Min (GI, WI) = Min (0.50, 0.75) = 0.50
Rule 6: Min (GI, WI) = Min (0.50, 0.00) = 0.00
Rule 7: Min (GI, WI) = Min (0.50, 0.25) = 0.25
Rule 8: Min (GI, WI) = Min (0.50, 0.75) = 0.50
Rule 9: Min (GI, WI) = Min (0.50, 0.00) = 0.00

### Aggregation

**Fig 13:**
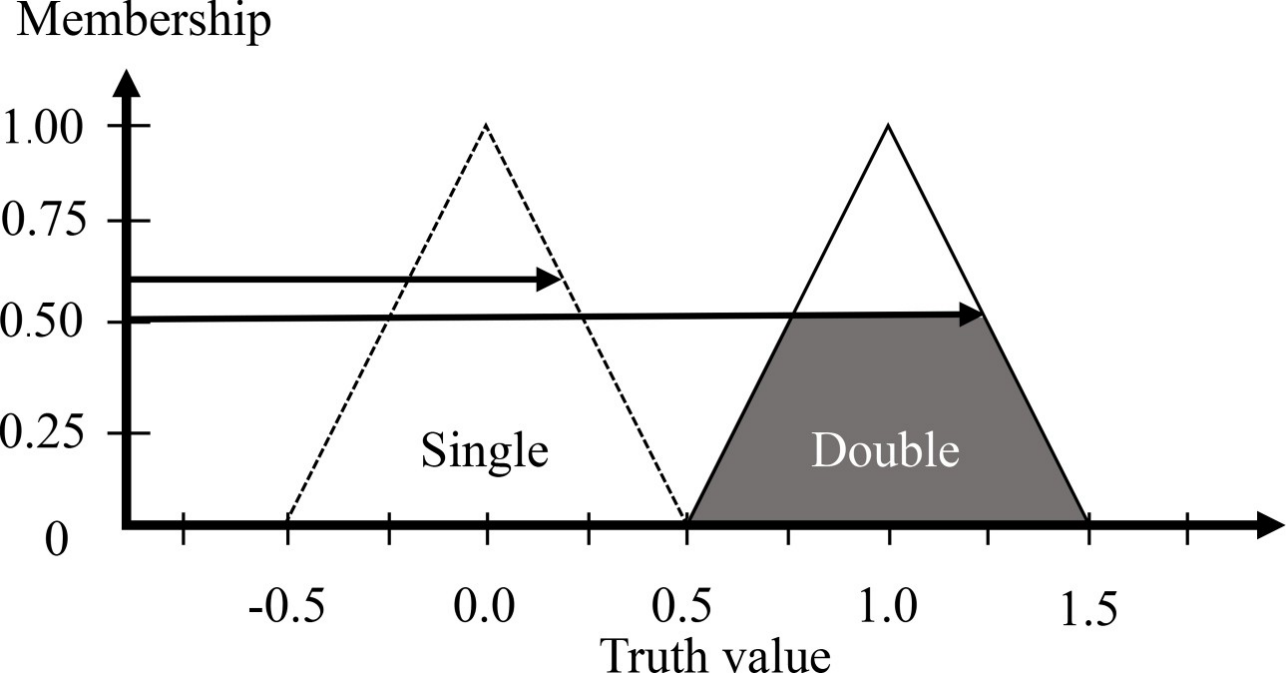
Result of output membership functions of the truth value.

Using the rule-based matrix by the root-sum-square, the degree of output memberships can be deduced (refer to Fig 13):

$$\begin{array}{l} False(Single)=\sqrt{{Rule}_{case1}^{2}+{Rule}_{case2}^{2}+{Rule}_{case4}^{2}+{Rule}_{case5}^{2}+{Rule}_{case7}^{2}}\\ \;=\sqrt{0.00_{case1}^{2}+0.00_{case2}^{2}+0.25_{case4}^{2}+0.50_{case5}^{2}+0.25_{case7}^{2}}\\ \;=0.612 \end{array}$$

$$\begin{array}{l} False(Double)=\sqrt{{Rule}_{case3}^{2}+{Rule}_{case6}^{2}+{Rule}_{case8}^{2}+{Rule}_{case9}^{2}}\\ \;=\sqrt{0.00_{case3}^{2}+0.00_{case6}^{2}+0.50_{case8}^{2}+0.00_{case9}^{2}}\\ \;=0.50 \end{array}$$

### Defuzzified

A fuzzy set is the output of each rule. The final result set is defuzzified, or fixed to a single number after the output fuzzy sets for all rules are combined into a single output fuzzy set of the control system. In this work, the fuzzy centroid of the combined area is measured by the center of gravity approach for defuzzification.

The most frequently used defuzzification method in the fuzzy controller is the centroid method. The defuzzified value, *d*_*CA*_ *(C)*, is defined as Eq (4).

$$d_{CA}(C)=\frac{\sum_{k=1}^{n}C(Z_{k})Z_{k}}{\sum_{k=1}^{n}C(Z_{k})}$$ (4)

where *C(z*_*k*_*)* is the result of the implication output, and *z*_*k*_ is the center value of each output.

Crispy output can be determined from the output membership function by calculating the fuzzy centroid of the area.

$$TruthValue=\frac{\left(False\times {Center}_{false})+(True\times {Center}_{true}\right)}{\left(False+True\right)}=\frac{\left(0.612\times 0)+(0.5\times 1\right)}{\left(0.612+0.50\right)}=0.449$$

The truth value is less than 0.50 that means the egg is a single yolk.

## Effect of the membership function shape of WI and GI

The membership function is an important factor for the accuracy of the system’s responses. However, to obtain the best function in each parameter is very difficult and needs a lot of data. The membership functions in this algorithm were created from a simple shape (triangular) with symmetry and non-symmetry that was mentioned in the previous section. This section is aimed to investigate the effects of the membership function shape on the accurate detection of double yolk eggs. The modification of the GI and WI membership functions was used in 20 cases. All cases were validated by experiments to detect any error. The error could be measured by comparing the results from the algorithms and experiment (by cracking the eggs). The single yolk error meant a cracked egg was single while the algorithm showed a double yolk egg. A double yolk error meant that the cracked egg was double while the algorithm showed a single yolk egg. The customers would then be satisfied when they bought double yolk eggs, and they received 100% double yolk eggs. Therefore, the best algorithm should be the minimum percentage of single yolk eggs error and the minimum percentage of a total error. Table 3 shows the shape of the GI and WI membership functions and their results.

**Table 3:** Shape of the input membership function and results.

Fuzzy No.	Geometry Indicator			Weight Indicator			Error (%)		
	Low	Medium	High	Low	Medium	High	SY-egg	DY-egg	Total
1	1.2000	1.3000	1.4000	50.0	70.0	90.0	24.37	0.72	25.09
2	1.2500	1.3500	1.4500	60.0	75.0	85.0	5.38	3.94	9.32
3	1.3000	1.4000	1.5000	65.0	75.0	85.0	1.43	8.24	9.68
4	1.2500	1.3500	1.5000	70.5	72.5	74.5	15.77	1.43	17.20
5	1.2750	1.3750	1.4750	69.0	74.0	79.0	9.68	4.66	14.34
6	1.2750	1.3625	1.4500	69.0	74.0	79.0	9.68	3.58	13.26
7	1.3400	1.3700	1.400	68.0	74.0	82.0	5.73	3.58	9.32
8	1.2750	1.3750	1.4750	70.5	72.5	74.5	15.77	1.43	17.20
9	1.3000	1.4000	1.4500	65.0	75.0	80.0	5.38	5.38	10.75
10	1.3500	1.3800	1.4000	68.0	77.0	80.0	4.66	5.02	9.68
11	1.3250	1.3675	1.4000	67.0	74.0	80.0	7.53	2.87	10.39
12	1.3250	1.3525	1.3850	69.5	74.0	79.5	8.96	2.15	11.11
13	1.3400	1.3500	1.3800	70.0	74.0	82.0	6.45	2.15	8.60
14	1.3400	1.3500	1.3800	69.0	76.0	82.0	5.38	3.94	9.32
15	1.3400	1.3800	1.4000	72.0	76.0	82.0	2.87	6.81	9.68
16	1.3400	1.3800	1.4000	69.0	75.0	81.0	5.38	3.58	8.96
17	1.3000	1.3500	1.3900	70.0	76.0	84.0	4.30	4.30	8.60
18	1.3000	1.3700	1.3800	72.0	73.0	75.0	15.41	2.51	17.92
19	1.3500	1.4000	1.5000	72.0	77.0	90.0	0.36	13.62	13.98
20	1.3000	1.3500	1.4500	65.0	73.0	83.0	6.09	2.87	8.96

From Table 3, the 13^th^ and 17^th^ cases showed minimum percentage of total error as 8.96% but percentage of single yolk egg error still high as 6.45% and 4.30%, respectively. While the 19^th^ case provided the minimum of single yolk egg error as 0.36% with high total error as 13.98%. Therefore, in this case, the algorithm of the 3^rd^ was used to program in the double yolk eggs detector prototype because it gives a low of single yolk error as 1.43% and a low total error as 9.68%. An algorithm to determine the optimum of GI and WI function to provide the minimum of all error does not perform in this research. The DY eggs detector prototype will mention in the next section.

## Developing the double yolk eggs detector prototype

**Fig 14:**
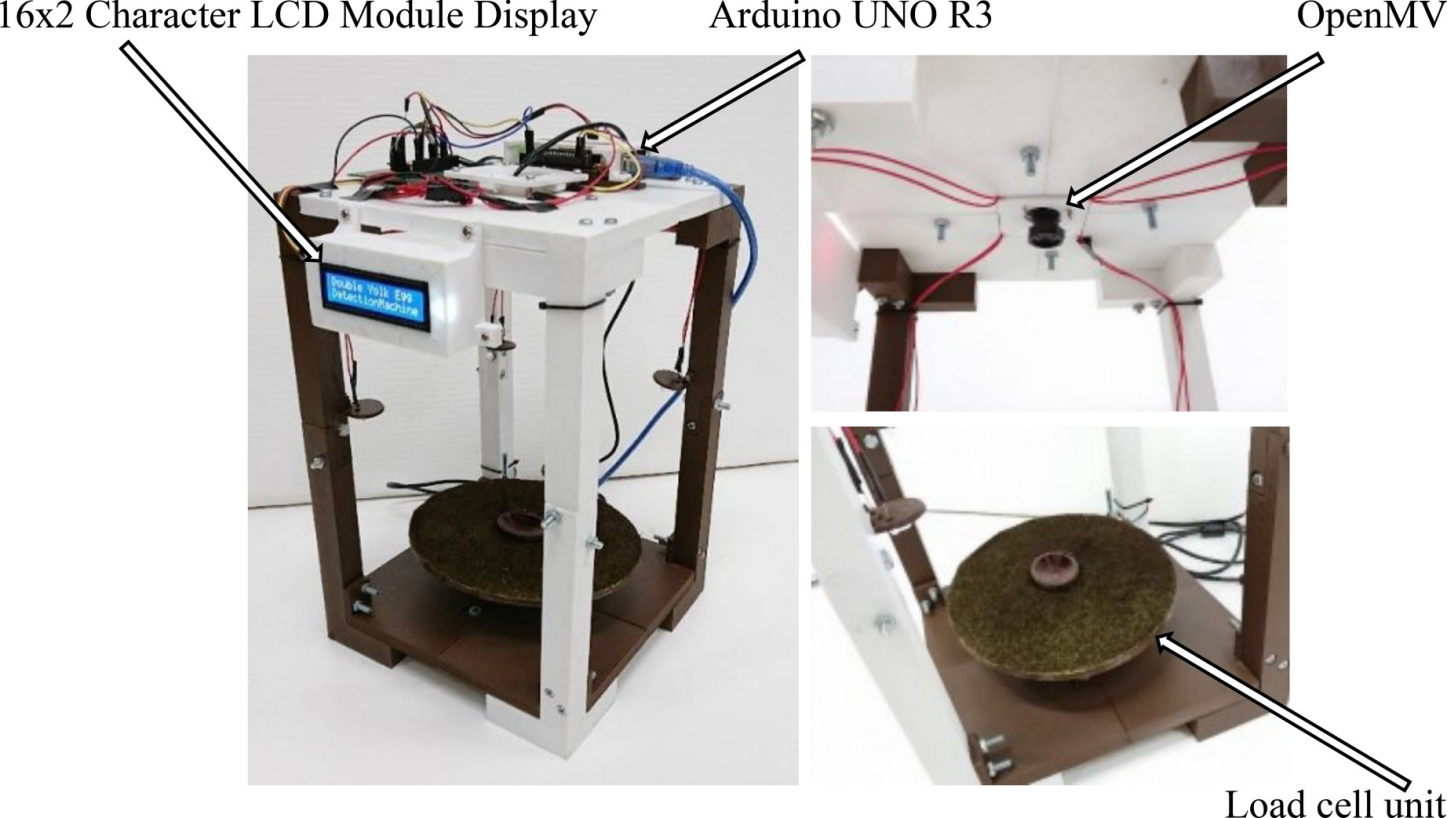
A prototype of double yolk egg detection.

In this research, the automated image process was developed to determine the geometric indicator of the eggs [15–17]; in other words, a major axis and a minor axis of a sample egg. The image was captured by a machine vision module powered by MicroPython. The eggs were placed on the base of the prototype machine with a green background to have a distinct background color from the egg. The prototype machine (refer to Fig 14) was assembled from 3D printed components using fused deposition modeling technology [18]. The top of the machine was installed with a machine vision module with the visibility to cover the egg, including the base. At the bottom of the machine was installed a load cell device connected to a microcontroller to read the weight and send the value to the machine vision module by means of the serial port.

**Fig 15:**
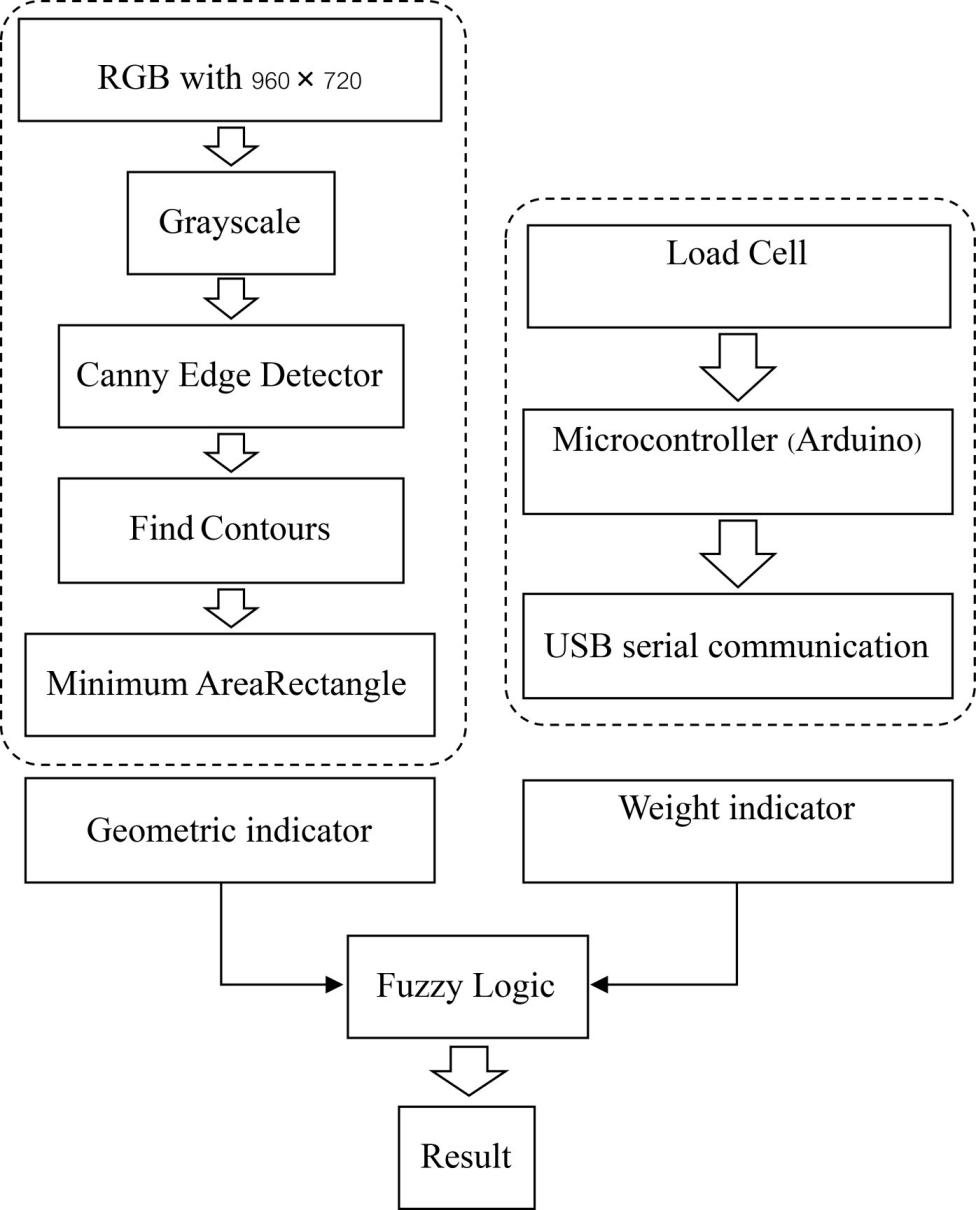
Procedure to develop a program for a double yolk eggs detector.

**Fig 16:**
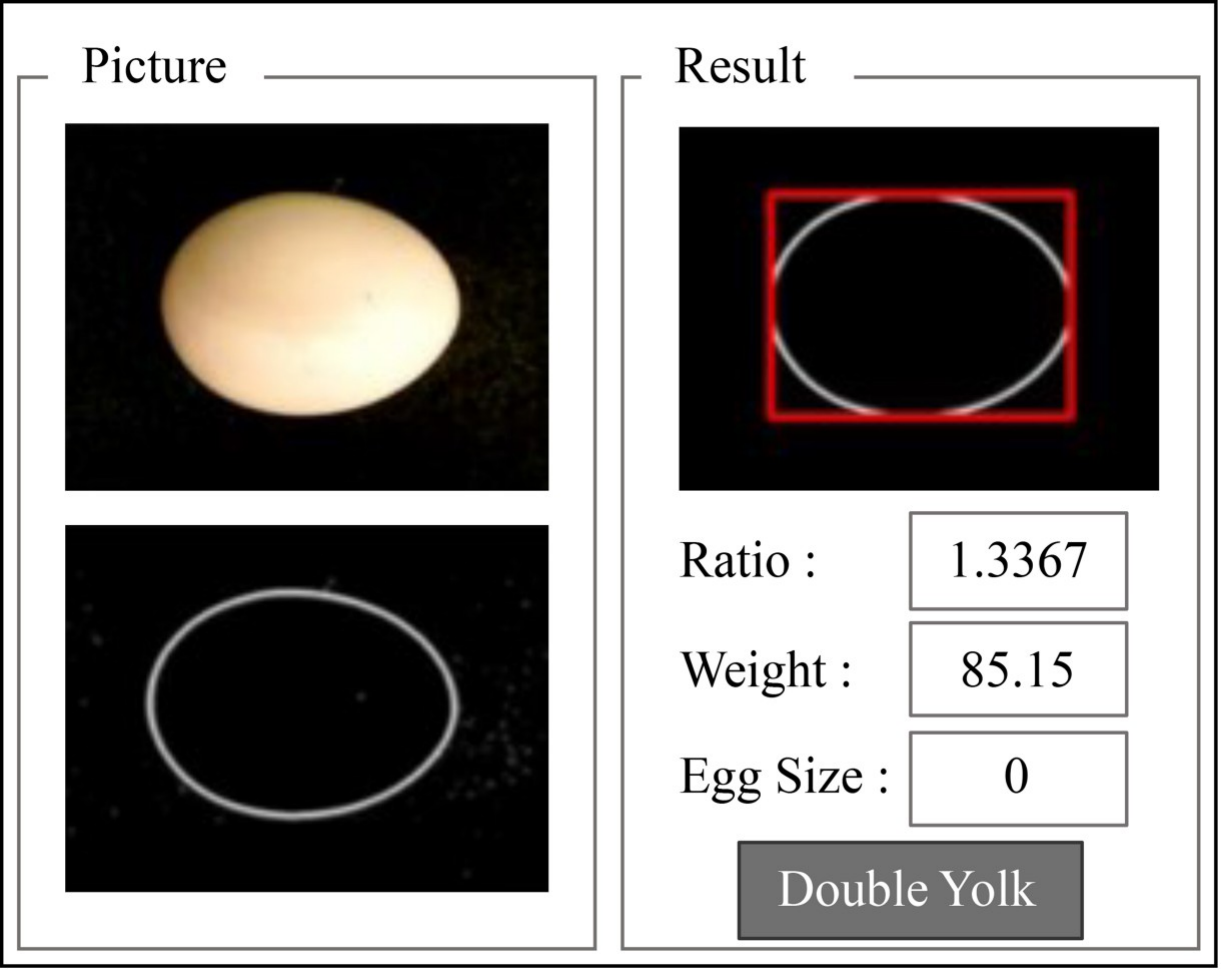
Interface of double yolk eggs detection.

The image processing began with receiving color images from the CCD sensor with a resolution of 960 × 720 pixels. The output image of the initial color image was a grayscale intensity image. By eliminating the saturation and hue data by staying with the luminance, the RGB image is converted to grayscale. Called a halftone image, the grayscale image darkened the shade of the initial image from black to white. Since the range of 8-bit intensity images with a grayscale colormap was only 256 shades of gray, equal to a full RGB color image, this halftone image offers better obtained data. The Canny edge detector is a technique to extract useful structural information from different vision was used to detect the edge of the egg [19]. After that, the edge of the image was searched for Contours using the Find Contours function [20]. As geometry indicators are a major axis and a minor axis of a sample egg, the researchers needed to find a rotated rectangle of the minimum area enclosing the contours of the egg to indicate the actual geometry of the egg by using the of the Minimum Area Rectangle Enclosing Method. The sequence of the processing to obtain the result is shown in the Fig 15 and the procedure to develop a program for a double yolk eggs detector is shown in the Fig 16. Compared to Wang’s method [11], the fuzzy logic detection of double yolks eggs was developed by using computer vision, which was required only normal visible light.

## Conclusion

The K-means technique is used for separating DY and SY by 2-dimension as weight and ratio of the eggs firstly, however the total error is still high as 15.05%. Fuzzy logic was applied to improve the error. The membership function of the weight and ratio of the egg were firstly defined by a simple with symmetry and asymmetry triangular function. The output was the truth value that meant the probability to detect double yolk eggs. The nine rules were conducted from experience and experimental data. The effect of GI and WI membership functions were investigated, the minimum percentage of the single yolk egg error and minimum percentage of total error are required. The triangular MF of weight as low = 65 g, medium = 75 g and high = 85 g, with triangular MF of ratio of the egg as low = 1.30, medium = 1.40 and high = 1.50 in the 3^rd^ case was provided a low of single yolk error as 1.43% and a low of total error as 9.68%. This error made both the customers and farmers satisfied. This function was selects to apply in the prototype of the DY detection machine prototype with Mbed platform.
